# Identification of Novel Linear Megaplasmids Carrying a ß-Lactamase Gene in Neurotoxigenic *Clostridium butyricum* Type E Strains

**DOI:** 10.1371/journal.pone.0021706

**Published:** 2011-06-28

**Authors:** Giovanna Franciosa, Concetta Scalfaro, Paola Di Bonito, Marco Vitale, Paolo Aureli

**Affiliations:** 1 Department of Food Safety and Veterinary Public Health, Istituto Superiore di Sanità, Rome, Italy; 2 Department of Infectious, Parasytic and Immunomediated Diseases, Istituto Superiore di Sanità, Rome, Italy; Max Planck Institute for Infection Biology, Germany

## Abstract

Since the first isolation of type E botulinum toxin-producing *Clostridium butyricum* from two infant botulism cases in Italy in 1984, this peculiar microorganism has been implicated in different forms of botulism worldwide. By applying particular pulsed-field gel electrophoresis run conditions, we were able to show for the first time that ten neurotoxigenic *C. butyricum* type E strains originated from Italy and China have linear megaplasmids in their genomes. At least four different megaplasmid sizes were identified among the ten neurotoxigenic *C. butyricum* type E strains. Each isolate displayed a single sized megaplasmid that was shown to possess a linear structure by ATP-dependent exonuclease digestion. Some of the neurotoxigenic *C. butyricum* type E strains possessed additional smaller circular plasmids. In order to investigate the genetic content of the newly identified megaplasmids, selected gene probes were designed and used in Southern hybridization experiments. Our results revealed that the type E botulinum neurotoxin gene was chromosome-located in all neurotoxigenic *C. butyricum* type E strains. Similar results were obtained with the 16S rRNA, the tetracycline *tet*(P) and the lincomycin resistance protein *lmr*B gene probes. A specific *mob*A gene probe only hybridized to the smaller plasmids of the Italian *C. butyricum* type E strains. Of note, a ß-lactamase gene probe hybridized to the megaplasmids of eight neurotoxigenic *C. butyricum* type E strains, of which seven from clinical sources and the remaining one from a food implicated in foodborne botulism, whereas this ß-lactam antibiotic resistance gene was absent form the megaplasmids of the two soil strains examined. The widespread occurrence among *C. butyricum* type E strains associated to human disease of linear megaplasmids harboring an antibiotic resistance gene strongly suggests that the megaplasmids could have played an important role in the emergence of *C. butyricum* type E as a human pathogen.

## Introduction

*Clostridium butyricum* is a butyric acid-producing anaerobic and spore-forming bacterium widely distributed in the environment and commonly present in the gut microflora of healthy humans and animals. It is generally considered a harmless saprophyte and it may be used for bioproduction of butyric acid, a widely applied food preservation and flavour-enhancing additive [1]. In addition, in certain countries, *C. butyricum* strains with proven absence of toxicological effects are allowed for use as probiotics in humans and animals, based on the beneficial effects that butyric acid and the bacteriocin(s) produced by those strains are believed to exert on the host intestine [2], [3].

On the other hand, an *in vitro* cytotoxic effect of butyric acid produced by *C. butyricum* has been demonstrated in various cells. This effect has been hypothesized to be responsible for the initiation of the intestinal lesions that lead to the mucosal necrosis of the ileum and colon observed in neonatal necrotizing enterocolitis, although conclusive evidence is still lacking [4], [5].

Most notably, some strains of *C. butyricum* have been described that are capable of synthesizing the type E botulinum neurotoxin (BoNT/E), which is one of the seven (A to G) antigenically distinct protein toxins that cause the flaccid paralysis of botulism. Neurotoxigenic *C. butyricum* type E strains were first isolated in Italy in 1984 from two distinct cases of infant botulism [6], an intestinal toxaemia by botulinum toxin-producing clostridia that affects babies under 1 year of age. Except for the ability to produce botulinum toxin, which is conventionally considered a distinctive characteristic of members of the *C. botulinum* species, these particular strains had all the phenotypic characteristics of the *C. butyricum* species [7]: their taxonomic identity was later genetically confirmed by DNA hybridization experiments and 16S rRNA gene sequencing [8]–[10]. Since then, neurotoxigenic *C. butyricum* type E has been implicated in further cases of infant botulism, as well as in several episodes of intestinal toxaemia in adults and of foodborne botulism (namely the classic intoxication caused by the ingestion of preformed BoNT in improperly preserved foods) [11]–[17]. Moreover, *C. butyricum* type E has been isolated from numerous lake sediments in China [18], where an outbreak of foodborne botulism had previously occurred [11]. Although neurotoxigenic *C. butyricum* type E has been rarely isolated up to now, it seems to be spread throughout the world, and in Italy it has been implicated in botulism cases more frequently than *C. botulinum* type E [19].

It is assumed that neurotoxigenic *C. butyricum* type E strains acquired the *bont*/E gene from a progenitor strain through mobile genetic elements [20]. Recently, the *bont*/A,/B and/F genes of certain *C. botulinum* strains were localized within circular plasmids [20]–[24], whereas the *bont*/G gene of *C. argentinense* has long been known to be plasmid-encoded [25], although whether the *bont*/G-encoding plasmid has a circular or linear structure remains unknown. Remarkably, some of the *bont*-encoding plasmids have been shown to be conjugative [26].

Previous studies have investigated the *bont*/E gene chromosomal or plasmid location in the genomes of neurotoxigenic *C. butyricum* type E. Although earlier experiments yielded discordant results, more recent studies indicated a chromosomal location for the *bont*/E gene in the neurotoxigenic *C. butyricum* type E isolates that had been analyzed [20], [27]–[29].

Here, we present evidence for the existence of previously unrecognized extremely large linear plasmids in ten neurotoxigenic *C. butyricum* type E strains isolated from clinical, food, and environmental sources in two widely separated geographical areas, Italy and China (Table 1). Given these unexpected findings, and because the genes encoding other BoNT types have been demonstrated to be chromosomally or plasmid located, depending on the clostridia strains being examined, the location of the *bont/*E gene in the genomes of the ten rare neurotoxigenic *C. butyricum* type E strains was re-evaluated. The genomic location of additional selected genes, including three different antibiotic resistance genes, was also determined in order to investigate the genetic content of the newly identified megaplasmids.

**Table 1:** Clostridia strains used in this study.

Strain	Source	Botulism	Country	Year of	Megaplasmid
				isolation	size (kb)^a^
Neurotoxigenic					
*C. butyricum* type E^b^					
ISS-20	Infant feces	Infant botulism	Italy	1984	>610
ISS-21	“	“	“	1985	>610
ISS-190	“	“	“	2001	∼825
ISS-86	Adult feces	Intestinal toxemia	Italy	1995	∼825
ISS-109	“	“	“	1996	> 610
ISS-145/1	“	Foodborne botulism	Italy	1999	> 610
LCL-063	“	“	China	1973	∼825
LCL-155	Food	“	“	1997	∼825
KZ-1886	Soil	−	China	1999	∼750–785^c^
KZ-1890	“	−	“	1999	∼750–785^c^
Non-neurotoxigenic					
*C. butyricum*					
ATCC 19398	ATCC	−	USA		−
UC-9035	Cheese	−	Italy		−
UC-9041	“	−	“		−
GP1	“	−	“		−
*C. botulinum*					
type E^d^					
CDC-4581	Adult feces	Foodborne botulism	USA		−
CDC-4234	“	“	“		−
CDC-5256	“	“	“		−

a The megaplasmid sizes were determined by comparison with a molecular standard (*S. cerevisiae* chromosomal DNA, Biorad).

b References 6, 11, 13-15, 18.

c Megaplasmid size in the range between 750 kb and 785 kb.

d Obtained from the culture collection of the Botulism Laboratory of the Center for Disease Control and Prevention, Atlanta, USA.

## Results

### Detection of mega-sized linear extrachromosomal bands in neurotoxigenic *Clostridium butyricum* type E isolates

**Figure 1:**
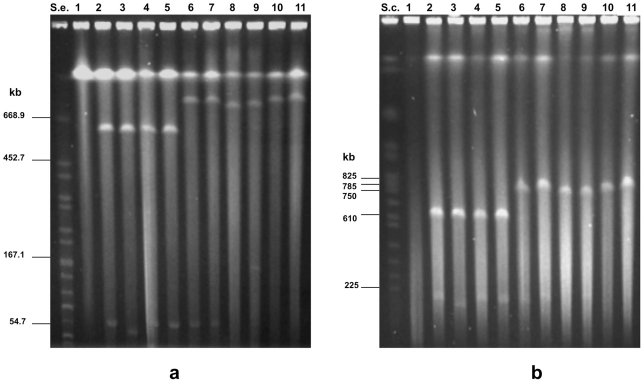
PFGE experiments showing the presence of an extrachromosomal mega-sized DNA band of > 600 kb in the undigested genomic DNA preparations from 10 neurotoxigenic *C. butyricum* type E strains: ISS-145/1 (lane 2); ISS-20 (lane 3); ISS-21 (lane 4); ISS-109 (lane 5); ISS-86 (lane 6); ISS-190 (lane 7); KZ-1886 (lane 8); KZ-1890 (lane 9); LCL-063 (lane 10); LCL-155 (lane 11). An additional extrachromosomal smaller band is evident in the DNA preparations from neurotoxigenic type E strains ISS-145/1, ISS-20, ISS-21, ISS-109, ISS-86, ISS-190 (lanes 2–7), and KZ-1890 (lane 9). Lane 1, undigested genomic DNA from non-neurotoxigenic *C. butyricum* strain ATCC 19398. S.e., *XbaI*-digested genomic DNA fragments of *Salmonella enterica* serotype Braenderup strain H9812 (ref. 35). S.c., *Saccharomyces cerevisiae* chromosomal DNA size marker (Biorad). PFGE conditions: 5–60 s pulse at 6 V/cm for 18 h (**1a**), and 50–90 s pulse at 6 V/cm for 20 h (**1b**). At the different PFGE conditions applied, the apparent sizes of the extrachromosomal mega-sized DNA bands (lanes 2–11) did not change in relation to the linear bands of the molecular size standards, consistent with the behaviour expected for linear DNA molecules. The mega-band sizes were estimated as follows (Figure 1b): >610 kb for strains ISS-20, ISS-21, ISS-109, ISS-145/1 (lanes 2–5); ∼825kb for strains ISS-86, ISS-190, LCL-063 and LCL-155 (lanes 6, 7, 10 and 11); and between 750 and 785 kb for strains KZ-1886 and KZ-1890 (lanes 8, 9). Migration of the extrachromosomal smaller DNA bands relative to the linear DNA markers was inconsistent (lanes 2–7 and lane 9 of Figures 1a and 1b), consistent with the behavior expected for circular super-coiled DNA molecules: as a consequence, the actual size of the extrachromosomal smaller bands of strains ISS-145/1, ISS-20, ISS-21, ISS-109, ISS-86, ISS-190 and KZ-1890 could not be determined.

Pulsed-field gel electrophoresis (PFGE) analysis of the undigested bacterial genomes has proven to be a valuable tool for demonstrating the existence of multiple replicons in bacteria [30], [31]. Our PFGE experiments revealed that the undigested genomic DNA preparations of each of ten neurotoxigenic *C. butyricum* type E strains contained a mega-sized DNA band of >600 kb in addition to the main chromosomal band (Figure 1). This result was apparently in contrast with the result previously obtained by Wang et al. [29], who detected by PFGE a single DNA chromosomal band in several neurotoxigenic *C. butyricum* type E strains, some of which were also analyzed in the present study (Table 1). However, the electrophoretic mobility of the DNA molecules is known to depend upon pulse times [32], and discrepancies between the above results are likely due to the different pulse time values used in the PFGE experiments: Wang *et al* used pulse times increasing from 3 to 20 s [29], whereas we used longer pulse times in different experiments (increasing from 5 to 60 s, or from 50 to 90 s) that allowed us to resolve the mega-sized extrachromosomal elements.

**Figure 2:**
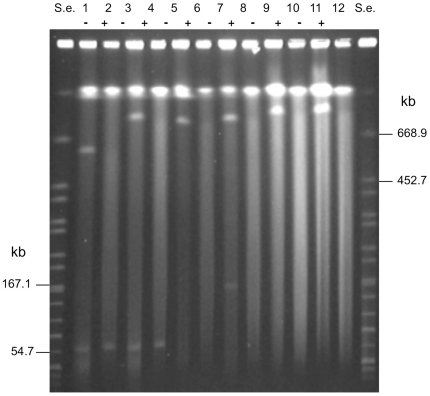
PFGE analysis of genomic DNA of six neurotoxigenic *C. butyricum* type E strains representative of the different megaplasmids sizes: ISS-109 (lanes 1 and 2); ISS-190 (lanes 3 and 4); KZ-1886 (lanes 5 and 6); KZ-1890 (lanes 7 and 8); LCL-063 (lanes 9 and 10); LCL-155 (lanes 11 and 12). S.e., *XbaI*-digested genomic DNA fragments of *Salmonella enterica* serotype Braenderup strain H9812 (ref. 35). PFGE conditions: 5–60 s pulse at 6 V/cm for 18 h. − and + indicate absence and presence of treatment with ATP-dependent exonuclease, respectively. The megaplasmid bands disappeared after treatment with the ATP-dependent nuclease, an enzyme that selectively digests linear DNA molecules, indicating that all megaplasmids had a linear structure (lanes 2, 4, 6, 8, 10, 12). The smaller plasmids of strains ISS-109 (lanes 1 and 2) and ISS-190 (lanes 3 and 4) were not susceptible to the ATP-dependent nuclease treatment, indicating that they had a circular structure. The ∼167 kb band of strain KZ-1890 disappeared after the ATP-dependent nuclease treatment (lanes 7 and 8), indicating that it was a linear DNA molecule; however, the same plasmid of strain KZ-1890 showed inconsistent PFGE migration typical of supercoiled DNA (lane 9 of Figures 1a and 1b). Hence, the smaller plasmid of strain KZ-1890 can exist in both linear and supercoiled forms, consistent with the behavior expected for circular DNA.

The high intensity of the mega-sized extrachromosomal bands after ethidium bromide staining indicated that they were present in multiple copies or that they might have a linear structure. The latter assumption is based on the different PFGE mobility of linear DNA molecules compared with that of large circular DNA molecules. Specifically, linear DNA molecules are totally released from the wells of PFGE gels and freely migrate through the gel according to their molecular size, whereas circular closed DNA molecules are in part retained within the wells of PFGE gels or they can partially migrate out of the wells in a supercoiled form, the mobility of which depends on changes in the applied pulse time values rather than on the molecular size, resulting in variation in the apparent sizes of the DNA molecules [33]. Our results supported the linear structure of the mega-sized extrachromosomal elements observed in the neurotoxigenic *C. butyricum* type E strains. First, the apparent sizes of the mega-bands did not change in relation to the linear bands of the molecular size standards when different pulse time conditions were used, consistent with the behavior expected for linear DNA (Figure 1). Second, the migration rate of the mega-sized extrachromosomal bands was not affected by treatment with S1 nuclease, an enzyme used to linearize circular DNA (data not shown). Finally, the mega-sized extrachromosomal bands were degraded by treatment with ATP-dependent exonuclease, an enzyme that selectively hydrolyzes linear DNA without affecting supercoiled and circular DNA (Figure 2), confirming that the extrachromosomal mega-bands had a linear structure.

At least four differently sized linear extrachromosomal bands ranging from ∼610 kb to ∼825 kb, as estimated by comparison with a molecular size standard (*S. cerevisiae* chromosomal DNA, Biorad), were detected in the ten neurotoxigenic *C. butyricum* type E strains examined in this study (Figure 1b). Specifically, a band greater than the 610-kb band of the molecular standard was observed in four Italian strains (ISS-20, ISS-21, ISS-109, ISS-145/1). A band approximately in line with the 825-kb band of the molecular size standard was observed in four strains, two from Italy (ISS-86 and ISS-190) and two from China (LCL-063 and LCL-155). The two remaining *C. butyricum* type E strains from China (KZ-1886 and KZ-1890) displayed slightly different bands ranging from 750 to 785 kb.

In addition to the mega-sized bands, the six neurotoxigenic *C. butyricum* type E isolates from Italy showed a smaller extrachromosomal band of an apparent size of ∼55 kb, whereas one of the strains from China (strain KZ-1890) displayed an additional extrachromosomal band, the apparent size of which was close to the 167.1-kb band of the molecular size standard (*XbaI*-digested genomic DNA fragments of *Salmonella enterica* serovar Braenderup strain H9812) [34] (Figure 1a). However, these smaller extrachromosomal bands disappeared from the gel after S1 nuclease treatment, indicating that they were susceptible to the enzyme activity (data not shown). In addition, the smaller plasmids of the Italian strains were not susceptible to the ATP-dependent nuclease treatment (Figure 2), indicating that they had a circular structure. The ∼ 167 kb band of strain KZ-1890 disappeared after the ATP-dependent nuclease treatment (Figure 2), indicating that it was a linear DNA molecule; however, since the same plasmid of strain KZ-1890 was susceptible to the S1 nuclease treatment and it showed an inconsistent PFGE migration (Figures 1a and 1b) typical of a supercoiled DNA molecule, it was concluded that this plasmid could exist in both linear and supercoiled forms, consistent with the behavior expected for circular DNA.

**Figure 3:**
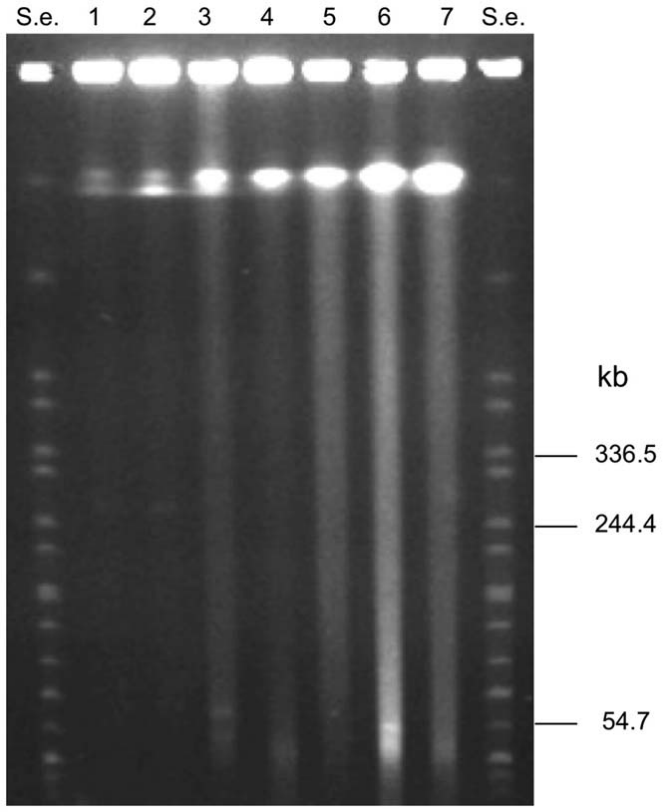
PFGE of undigested genomic DNA of non-neurotoxigenic *C. butyricum* strains (lanes 1–4) and *C. botulinum* type E strains (lanes 5–7). Strains: UC-9035 (lane 1); UC-9041 (lane 2); GP1 (lane 3); ATCC 19398 (lane 4); CDC-5234 (lane 5); CDC-4581 (lane 6); CDC-5380 (lane 7). S.e., *XbaI*-digested genomic DNA fragments of *Salmonella enterica* serotype Braenderup strain H9812 (ref. 35). PFGE conditions: 5–60 s pulse at 6 V/cm for 18 h. Extrachromosomal bands close to the 54.7 kb band of the molecular standard (S.e.) are evident in lane 3 (non-neurotoxigenic *C. butyricum* strain GP1) and lane 6 (*C. botulinum* type E strain CDC-4581).

PFGE of the undigested genomic DNA obtained from the non-neurotoxigenic *C. butyricum* strains and from the *C. botulinum* type E strains included in this study (Table 1) either showed no extrachromosomal bands or extrachromosomal bands much smaller than the mega-sized ones observed in the neurotoxigenic *C. butyricum* type E isolates (Figure 3).

**Figure 4:**
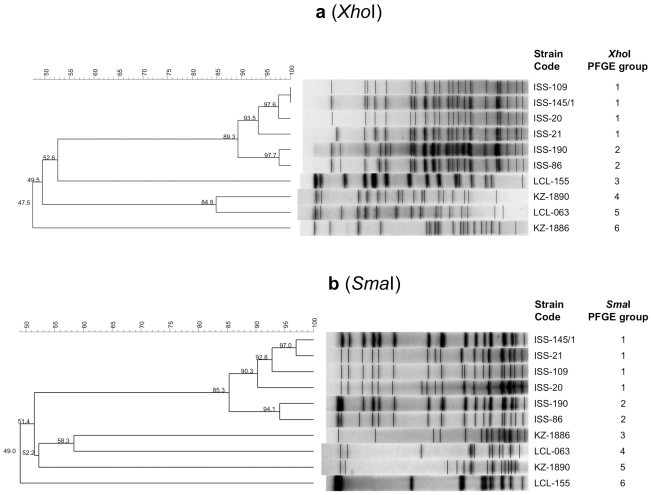
Clustering analysis of the PFGE profiles of the ten neurotoxigenic *C. butyricum* type E strains obtained by *Xho*I (4a) and *Sma*I (4b) endonucleases. The similarity of PFGE profiles was evaluated using the Bionumerics software (version 4.0) (Applied Maths, Sint-Martens-Latem, Belgium); a similarity level of ≥ 90% was used to define PFGE groups. The 10 neurotoxigenic *C. butyricum* type E strains were divided in 6 PFGE groups with both endonucleases.

Analysis of the *Xho*I and *Sma*I PFGE macrorestriction profiles of the genomic DNA from the neurotoxigenic *C. butyricum* type E strains revealed that the four Chinese strains clustered into four different PFGE groups with both endonucleases, and the six Italian strains clustered into two major PFGE groups with an intra-linkage homology level of ≥90% with both *Xho*I and *Sma*I endonucleases (Figure 4). Each of the PFGE groups was consistent with a different size of mega-sized extrachromosomal bands observed among the strains.

### *Bont*/E gene carriage and copy number

**Figure 5:**
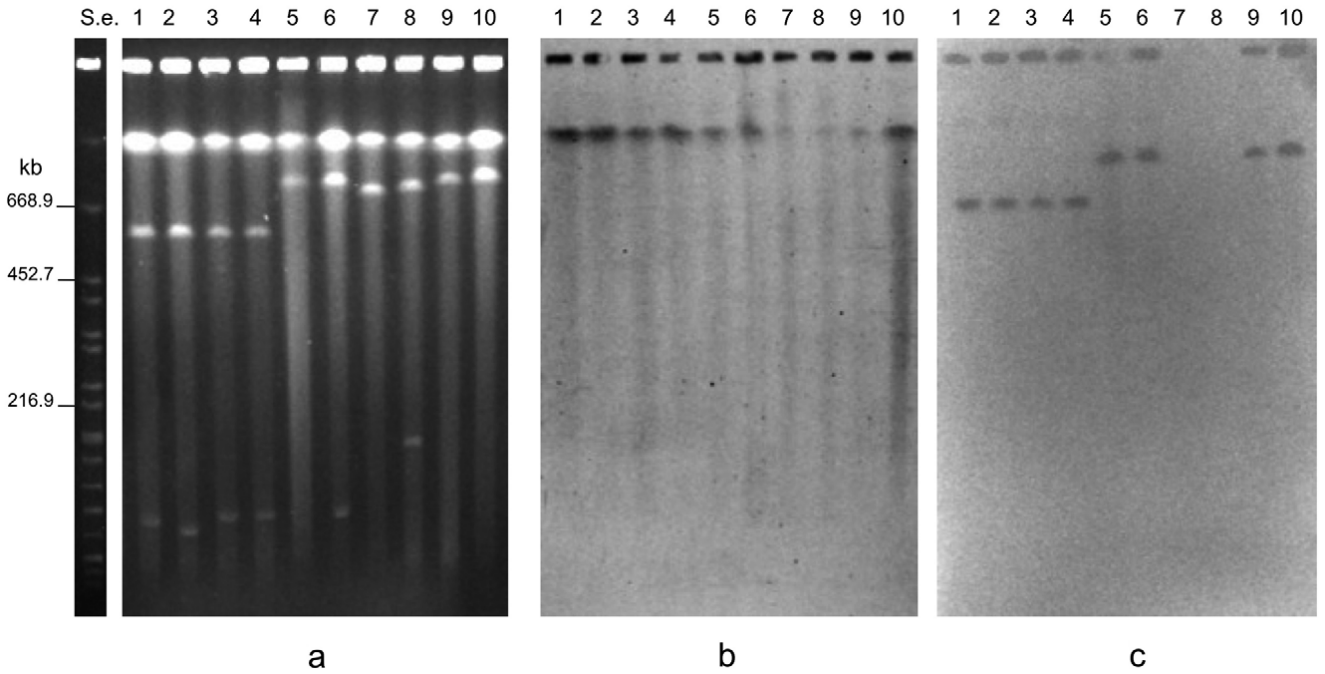
PFGE of undigested genomic DNA of strains ISS-145/1 (lane 1); ISS-20 (lane 2); ISS-21 (lane 3); ISS-109 (lane 4); ISS-86 (lane 5); ISS-190 (lane 6); KZ-1886 (lane 7); KZ-1890 (lane 8); LCL-063 (lane 9); LCL-155 (lane 10). S.e., *XbaI*-digested genomic DNA fragments of *Salmonella enterica* serotype Braenderup strain H9812 (ref. 34). PFGE conditions: 5–60 s pulse at 6 V/cm for 18 h (**5a**). Southern hybridization with a *bont*/E gene probe showing that the gene probe hybridized to the chromosome bands of strains (**5b**). Southern hybridization with a β-lactamase gene probe showing that the gene probe hybridized to the megaplasmids of strains ISS-145/1 (lane 1); ISS-20 (lane 2); ISS-21 (lane 3); ISS-109 (lane 4); ISS-86 (lane 5); ISS-190 (lane 6); LCL-063 (lane 9); LCL-155 (lane 10) (**5c**).

Southern-blot analysis following PFGE of the undigested DNA of the neurotoxigenic *C. butyricum* type E strains with a specific *bont*/E gene probe revealed an hybridization signal corresponding to the chromosomal bands of all strains (Figure 5b).

**Figure 6:**
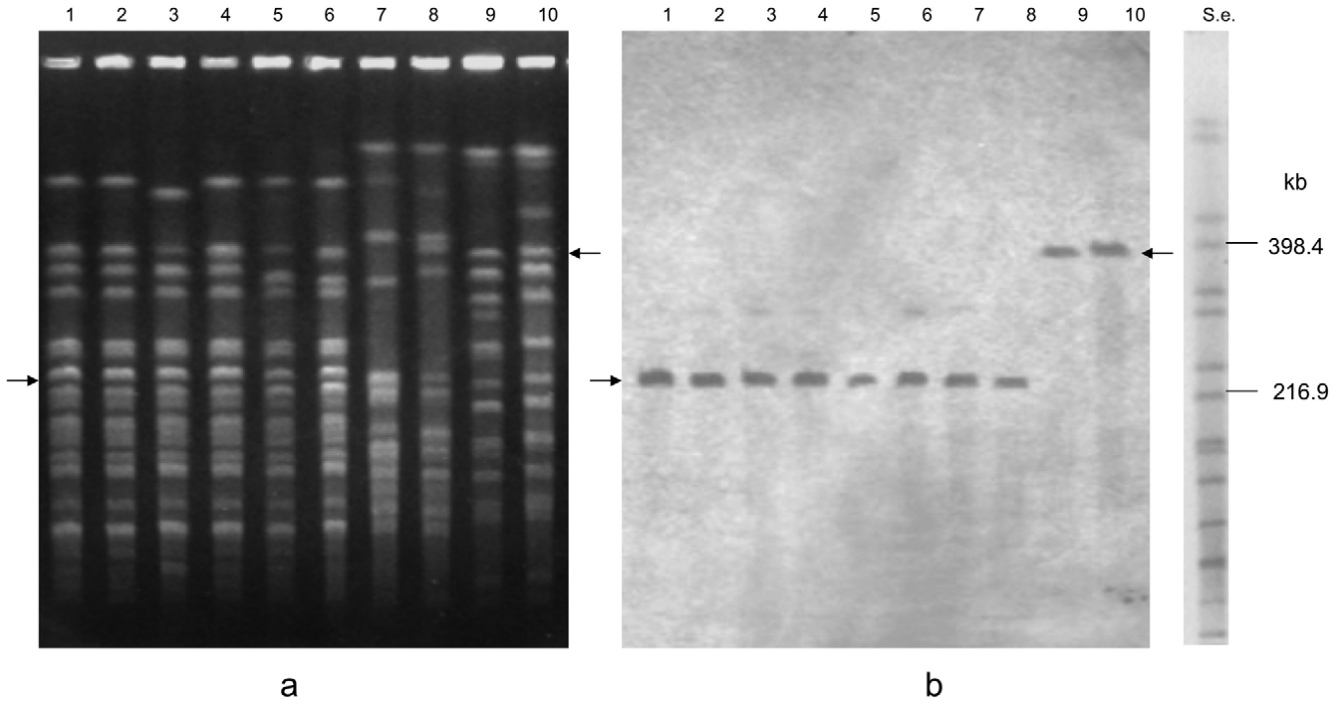
PFGE of *Xho*I-digested DNA of strains ISS-145/1 (lane 1); ISS-20 (lane 2); ISS-21 (lane 3); ISS-109 (lane 4); ISS-86 (lane 5); ISS-190 (lane 6); KZ-1886 (lane 7); KZ-1890 (lane 8); LCL-063 (lane 9); LCL-155 (lane 10). S.e., *XbaI*-digested genomic DNA fragments of *Salmonella enterica* serotype Braenderup strain H9812 (ref. 34). PFGE conditions: 4–40 s pulse at 6 V/cm for 18 h (**6a**). Southern hybridization with a *bont*/E gene probe showing that the gene probe hybridized to single restriction bands, as indicated by the black arrows (**6b**).

The *Xho*I- and *Sma*I-digested DNA samples of the neurotoxigenic *C. butyricum* type E strains were also hybridized with the *bont*/E gene probe to determine the copy number of the *bont*/E gene. The results showed a single hybridization band in all analyzed neurotoxigenic *C. butyricum* type E strains, indicating that they all contained a single copy of the *bont*/E gene (Figure 6).

### Carriage of 16S rRNA and *mob*A genes

The genomic location of the 16S rRNA genes was investigated to determine whether the mega-sized extrachromosomal bands were plasmids or second chromosomes; by convention, the presence of ribosomal RNA genes is indicative of a chromosome because such essential genes of the translational machinery are generally not carried within plasmids in bacteria [35]. The specific 16S rDNA gene probe that we used only hybridized to the chromosomal bands of the ten neurotoxigenic *C. butyricum* type E strains (data not shown), indicating that the extrachromosomal bands were indeed mega-plasmids rather than second chromosomes.

**Figure 7:**
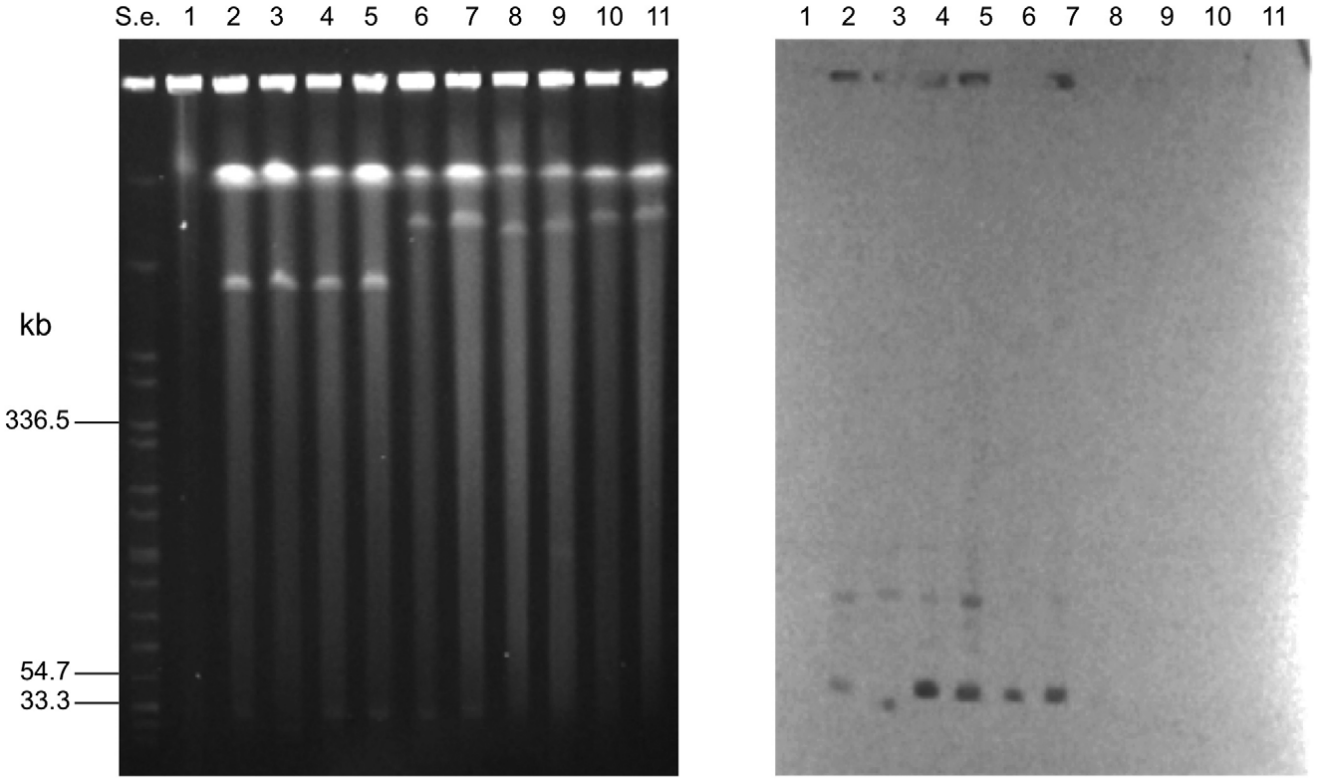
PFGE of undigested genomic DNA of strains ATCC 19398 (lane 1); ISS-145/1 (lane 2); ISS-20 (lane 3); ISS-21 (lane 4); ISS-109 (lane 5); ISS-86 (lane 6); ISS-190 (lane 7); KZ-1886 (lane 8); KZ-1890 (lane 9); LCL-063 (lane 10); LCL-155 (lane 11) (7a). S.e., *XbaI*-digested genomic DNA fragments of *Salmonella enterica* serotype Braenderup strain H9812 (ref. 35). PFGE conditions: 5–60 s pulse at 6 V/cm for 18 h. Southern hybridization with a specific *mobA* gene probe showing that the gene probe hybridized to the smaller plasmids observed in lanes 2–7 (strains ISS-145/1, ISS-20, ISS-21, ISS-109, ISS-86, ISS-190) (**7b**).

BLAST comparison between the draft whole-genome sequences of two neurotoxigenic *C. butyricum* type E strains (accession numbers NZ_ACOM00000000 for strain BL5262 that corresponds to strain ISS-20 of this study, and NZ_ABDT00000000 for strain 5521 that corresponds to strain ISS-21 of this study) and a ∼8-kb bacteriocin-encoding plasmid from a non-neurotoxigenic *C. butyricum* strain (pCBM588, accession number NZ_ABDT00000000; the only available complete DNA sequence from a non-neurotoxigenic *C. butyricum* strain) [36], showed a high percentage of nucleotide identity (93%) in a 2-kb DNA region. This region included a gene for a mobilization protein of the MobA/MobL family that is essential for specific plasmid transfer [37] **.** A specific *mob*A gene probe that we designed only hybridized to the smaller extrachromosomal bands observed in the Italian *C. butyricum* type E strains (Figure 7).

### Carriage of antibiotic resistance genes

Several antibiotic resistance genes were present in both draft genome sequences of the Italian neurotoxigenic *C. butyricum* type E strains. As antibiotic resistance genes are often found within bacterial plasmids, we investigated the location of representative antibiotic resistance genes (i.e., the tetracycline *tet*(P) gene, the lincomycin resistance protein *lmr*B gene, and a ß-lactamase gene) in the genomes of the ten neurotoxigenic *C. butyricum* type E strains. The *tet*(P) gene probe that we used hybridized to the chromosomes of all ten strains that were analyzed. The *lmr*B gene probe hybridized to the chromosomes of the six strains isolated in Italy, whereas no hybridization signal was observed for the four strains from China (data not shown), indicating that only the Italian strains contained the *lmr*B gene. Finally, the ß-lactamase gene probe hybridized to the megaplasmid bands of eight of the ten neurotoxigenic *C. butyricum* type E strains (Figure 5c), of which six from Italy and two from China (LCL-063 and LCL-155); the two remaining Chinese strains (KZ-1886 and KZ-1890) did not show any hybridization signal with the ß-lactamase gene probe that we used, indicating that they did not harbor that ß-lactamase gene.

## Discussion

In this study, we show the presence of a linear megaplasmid in the genomes of ten neurotoxigenic *C. butyricum* type E strains with different clinical and geographical origins. The linear megaplasmids displayed size heterogeneity, with at least four different sizes being recognized. The differently sized megaplasmids were distributed among the *C. butyricum* type E strains according to the distinct PFGE groups they belonged to, indicating that the megaplasmids likely contributed to the clonal diversity. Besides, some of the analyzed strains were shown to possess additional smaller circular plasmids. These unprecedented findings indicate that neurotoxigenic *C. butyricum* type E strains share a complex genomic organization, with multiple linear and circular replicons, thus providing new insights into the genetics of these microorganisms.

The occurrence of plasmids has already been reported in several *Clostridium* spp, including *C. perfringens*, *C. difficile*, *C. tetani, C. argentinense* and *C. botulinum* [38]. However, to our knowledge, the largest plasmid described so far in the clostridia genus is a 270-kb circular plasmid (pCLJ, Accession Number NC_012654) that simultaneously harbors two *bont* genes, the *bont*/A and/B genes [22]. This plasmid is considerably smaller than the megaplasmids of 600 kb to 850 kb that are described here. DNA molecules of these huge sizes have largely remained undetected due to the considerable technical challenges that their separation from the chromosomes involves. Considering that the sizes of two neurotoxigenic *C. butyricum* type E genomes have been estimated to be ∼4.5 Mb and 4.7 Mb [20], the megaplasmids would constitute 13–19% of the whole bacterial genome. Such genome sizes are larger than all *C. botulinum* genomes available to date in the GenBank database, which have reported sizes of 2.3 Mb to 4.2 Mb, in line with the presence of megaplasmids in the genomes of neurotoxigenic *C. butyricum* type E strains. However, comparison with the genome sizes of non-neurotoxigenic *C. butyricum* is not possible because genome sequencing data are lacking for that organism.

Furthermore, our results indicate that the megaplasmids identified in the neurotoxigenic *C. butyricum* type E strains have a linear structure. Bacterial linear plasmids cannot readily be recognized by genome sequencing owing to the inability to generate a closed circular DNA sequence; however, they can be identified by their migratory behaviour under different PFGE conditions and by the use of specific nucleases that selectively cut linear or circular DNA molecules. Although linearization of circular plasmids may occur due to cell death and/or during DNA extraction, the concomitant presence in some of the tested *C. butyricum* type E strains of additional smaller plasmids that were not linearized under the same experimental conditions provided an internal control for the absence of DNA breakage, indicating that the megaplasmids were native linear DNA molecules and not simple artefacts.

Linear plasmids have been established in both Gram-positive and Gram-negative bacteria, including *Streptomyces* spp, *Mycobacterium* spp, *Bacillus* spp, *Borrelia* spp, *Lactobacillus* spp, *Rhodococcus* spp, *Micrococcus* spp, *Escherichia coli*, *Salmonella enterica*, *Klebsiella oxytoca*, *Yersinia enterocolitica*, as well as in some eukaryotic microorganisms [39]. However, no linear plasmids have yet been reported in *Clostridium* spp.

In general, comparative genomic studies have indicated that bacteria with large composite genomic structures might be more ecologically successful in certain environments [40]. In particular, both linear and circular plasmids are usually assumed to confer a selective advantage to the host, by providing an additional repertoire of genes that significantly extend the metabolic versatility, fitness and stress resistance of the microorganism, even though cryptic linear and circular plasmids also exist. Our attempts to localize within the newly recognized linear megaplasmids some of the genes important for the biology of neurotoxigenic *C. butyricum* type E, including the *bont*/E-encoding gene, a plasmid mobilization protein gene, and two antibiotic resistance genes (*tet*(P) and *lmr*B genes) were unsuccessful. However, a major finding of this study was that the megaplasmids of eight of the ten analyzed neurotoxigenic *C. butyricum* type E strains carried a gene coding for a ß-lactamase, an enzyme that hydrolyzes the β-lactam class of antibiotics such as penicillins and cephalosporines. Interestingly, most of the *C. butyricum* type E strains that contained the megaplasmid-encoded ß-lactamase gene had originally been isolated from the clinical specimens of people affected with intestinal toxaemia botulism (five strains) or foodborne botulism (two strains). Thus, it is likely that the neurotoxigenic *C. butyricum* type E strains harbouring the megaplasmid-encoded β-lactamase gene might have a selective advantage in a clinical setting. Although antibiotics are not recommended for treating intestinal toxaemia botulism and foodborne botulism, their use may be necessary to control secondary infections [41]; β-lactam antibiotics are among the most commonly prescribed antimicrobial drugs, thus inducing selective pressure for resistance genes. Both neurotoxigenic *C. butyricum* type E strains whose megaplasmids did not hybridize to the ß-lactamase gene probe were from environmental sources (Chinese soil samples): whether the megaplasmids of those strains carry antibiotic resistance genes other than the β-lactamase gene remains to be determined.

The ß-lactamase probe that hybridized to the megaplasmids of eight *C. butyricum* type E strains was located at 279338–279745 within contig 1 (NZ_ACOM01000001.1) of the draft genome sequence of strain BL5262 (ISS-20 of the present study). Hence, the megaplasmid of strain ISS-20, whose size was estimated >610 kb by comparison with a molecular marker in the present study, is likely (part of) this contig (757,653 bp). Interestingly, another ß-lactamase-encoding gene, a gene coding for a metallo-ß-lactamase family protein, and a ß-lactamase domain protein gene are also present within contig 1. Besides, this contig contains other important putative genes, including the α-subunit of a DNA polymerase III (Gram-positive type) gene, the nitrogenase operon that is absent from the other sequenced contigs, genes coding for the phosphotransferase (PTS) systems for the import of sugars, genes reminiscent of phage proteins such as a phage membrane protein and a recombinase of the phage integrase family, and the genes encoding the CRISPR-associated Cas proteins involved in the acquired immunity against viruses and plasmids (a system evocative of the eukaryotic RNA interference system), several membrane sensor proteins and ATP-binding transporter (ABC transporter) of various substrates.

It will be interesting to analyze and compare the gene contents of the other *C. butyricum* type E megaplasmids identified in the present study, specially those that are greater than the one of ISS-20 strain, in order to understand which genes are conserved within the megaplasmids and which ones have more recently been acquired.

In conclusion, the widespread occurrence of linear megaplasmids in the neurotoxigenic *C. butyricum* type E isolates, and their absence from non-neurotoxigenic *C. butyricum* and *C. botulinum* type E isolates, would suggest that they are essential for the survival and growth of the former microorganisms. The fact that the megaplasmids of neurotoxigenic *C. butyricum* type E strains associated with human botulism carry a β-lactam resistance gene could have contributed to the emergence of neurotoxigenic *C. butyricum* type E as a human pathogen. Research on the role of the megaplasmids in the spread of antibiotic resistance among clostridia and/or other microorganisms is warranted. We believe that findings reported in the present study can give new impulse for completing the genome sequences of the neurotoxigenic *C. butyricum* type E strains.

## Materials and Methods

### Clostridia strains

The clostridia strains used in this study are listed in Table 1. Ten strains were neurotoxigenic *C. butyricum* type E. Of these, six had been isolated in Italy from as many patients suffering either from intestinal toxaemia botulism (ISS-20, ISS-21, ISS-86, ISS-109, ISS-190) or food-borne botulism (ISS-145/1) [6], [13]–[15]: they were obtained from the ISS culture collection. The other four *C. butyricum* type E strains were from food (LCL-155) and human (LCL-063) specimens isolated from two different food-borne botulism outbreaks in China, and from soil samples (KZ-1886 and KZ-1890) collected from the areas where the outbreaks had occurred [11], [18]: they were a kind gift from Professor Nakamura, Kanazawa University, Japan.

All strains had genetically been confirmed as *C. butyricum* [8], [9], [42]; however, they all produced BoNT/E and carriage of the corresponding *bont*/E gene had previously been shown by PCR [13], [15], [29], [43]. In addition, four non-neurotoxigenic *C. butyricum* strains and three *Clostridium botulinum* type E strains were included in the experiments (Table 1).

All clostridia strains were stored at −80°C in microbank cryogenic vials (Prolab Diagnostics, Austin, TX). Clostridia stock cultures were grown for 48 h at 37°C on egg yolk agar (EYA) plates (Oxoid, Milan, Italy) under anaerobiosis (GasPack jars, Oxoid). For growth of broth cultures, TPGY broth (5% Trypticase, 0.5% peptone, 0.4% glucose, 2% yeast exctract, 1% L-cysteine hydrocloride monohydrate) was used.

### Pulsed-field gel electrophoresis (PFGE)

Overnight TPGY cultures of the clostridia strains were used for DNA preparation. Cells were embedded in 1.5% low-melting-point agarose (Invitrogen, Carlsbad, CA, USA) plugs and genomic DNA was extracted as described previously [23]. Some DNA plugs were treated with S1 nuclease (MBI Fermentas, Lithuania) as detailed elsewhere [23]. Other DNA plugs were digested with ATP-dependent exonuclease (Epicentre Technologies, Madison, WI) according to the manufacturer's instructions. Restriction of the DNA plugs with *Xho*I or *Sma*I (New England BioLabs, Ipswich, MA) was performed under conditions recommended by the manufacturers.

Undigested and enzymatically treated genomic DNA samples were separated in a contour-clamped homogeneous electric field system (CHEF Mapper apparatus, BioRad Laboratories, Hercules, CA) through 0.8% Seakem Gold agarose gel (Cambrex, East Rutherford, NJ) in 0.5 X Tris-borate-EDTA buffer. Electrophoresis was performed at 6 V/cm and 14°C. The pulse time increased from 5 to 60 s (linear ramping factor) over 18 h. The DNA isolated from Salmonella *enterica* serovar Braenderup strain H9812 and restricted with *Xba*I (New England BioLabs, Ipswich, MA) was used as the molecular marker [34]. In some experiments, the undigested DNA samples were separated by increasing the pulse time from 50 to 90 s over a 20 h run, to more accurately estimate the sizes of the DNA molecules; when these parameters were applied, *Saccharomyces cerevisiae* chromosomal DNA (Biorad) was used as molecular marker.

Gels were stained with ethidium bromide and visualized using a GelDoc 2000 apparatus (Bio-Rad). The PFGE restriction profiles were compared using Bionumerics software (version 4.0) (Applied Maths, Sint-Martens-Latem, Belgium). Clustering was performed by applying the Dice coefficient and the unweighted pair-group method using arithmetic averages (UPGMA), with 1% optimization and 1% position tolerance.

### Southern-blot analyses

Digoxigenin (DIG)-labeled probes were generated by a PCR DIG Probe Synthesis kit (Roche Diagnostics GmbH, Mannheim, Germany). Primers for the preparation of probes from the *bont*/E, 16SrRNA, mobilization protein (*mob*A), tetracycline *tet*(P), *lmr*B efflux, and ß-lactamase genes are described in Table 2 and were purchased from Eurofins MWG (Ebersberg, Germany). Southern hybridizations of pulsed-field gels with the gene probes were performed as previously described [23]. DIG detection reagents were obtained from Roche Diagnostics. The CSPD substrate (Roche Diagnostics) was used for detection of hybridized probes according to the manufacturer's instructions.

**Table 2:** Primers used to generate the specific gene probes for the Southern blot experiments.

Gene primers	Sequence (5′→ 3′)	Amplicon size (bp)
*cnt*E		508
forward	AATGGGAGCAGAGCCTGATTT	
reverse	TACCGAATAAATTCCGCTAGC	
ß-*lactamase* 1		408
forward	ATGGGGAGAACGTCATAC	
reverse	TTGCCGTCATAGTGAGGT	
*16SrRNA*		392
forward	TAGATACCCTGGTAGTCCACG	
reverse	GATGATTTGACGTCATCCCCA	
*mob*A		911
forward	CTAATGAATTGACCTCTCTAC	
reverse	TGCTCATGTAATGCTGACTAT	
*tet(*P)		372
forward	AGTAAGTGCAGCAGAAGGTGT	
reverse	TCATCCTGAAGAGCACATCCT	
*lmr*B		417
forward	AATCCAGAAGCAACTGCACTC	
reverse	TTCACAGCTGCTATGGCACTT	

1 ß-*lactamase* gene located at nucleotides 278991–280007 within contig 1 (NZ_ACOM01000001.1) of the draft genome sequence of strain BL5262.
